# Investigation of Islet2a function in zebrafish embryos: Mutants and morphants differ in morphologic phenotypes and gene expression

**DOI:** 10.1371/journal.pone.0199233

**Published:** 2018-06-21

**Authors:** Rosa L. Moreno, Kristina Williams, Kenneth L. Jones, Angeles B. Ribera

**Affiliations:** 1 Department of Physiology and Biophysics, University of Colorado School of Medicine, Aurora, Colorado, United States of America; 2 Department of Pediatrics, University of Colorado School of Medicine, Aurora, Colorado, United States of America; National University of Singapore, SINGAPORE

## Abstract

Zebrafish primary motor neurons differ from each other with respect to morphology, muscle targets and electrophysiological properties. For example, CaP has 2-3-fold larger densities of both inward and outward currents than do other motor neurons. We tested whether the transcription factor Islet2a, uniquely expressed in CaP, but not other primary motor neurons, plays a role in specifying its stereotypic electrophysiological properties. We used both TALEN-based gene editing and antisense morpholino approaches to disrupt Islet2a function. Our electrophysiology results do not support a specific role for Islet2a in determining CaP’s unique electrical properties. However, we also found that the morphological phenotypes of CaP and a later-born motor neuron differed between *islet2a* mutants and morphants. Using microarrays, we tested whether the gene expression profiles of whole embryo morphants, mutants and controls also differed. Morphants had 174 and 201 genes that were differentially expressed compared to mutants and controls, respectively. Further, *islet2a* was identified as a differentially expressed gene. To examine how mutation of *islet2a* affected *islet* gene expression specifically in CaPs, we performed RNA *in situ* hybridization. We detected no obvious differences in expression of *islet1*, *islet2a*, or *islet2b* in CaPs of mutant versus sibling control embryos. However, immunolabeling studies revealed that an Islet protein persisted in CaPs of mutants, albeit at a reduced level compared to controls. While we cannot exclude requirement for some Islet protein, we conclude that differentiation of the CaP’s stereotypic large inward and outward currents does not have a specific requirement for Islet2a.

## Introduction

Mammalian spinal motor neurons comprise a heterogeneous population, as evidenced by their different morphological and functional properties [1] [2]. Several lines of evidence support the view that different combinations of LIM-homeodomain (LIM-HD) transcription factors direct specification of the diverse set of mammalian motor neuron subtypes [3][4].

In the zebrafish spinal cord, early born primary motor neurons (PMNs) also are heterogeneous and express LIM-HD transcription factors in a combinatorial manner [5–10]. Each hemisegment has three different PMNs—RoP, MiP, CaP; in some hemisegments, there is also a variably-present PMN, VaP, a CaP duplicate that dies early [11]. PMNs differ from each other with respect to gene expression, soma position, axonal trajectory and/or electrical membrane properties [5,8 12]. At the time of axon genesis, CaP expresses *islet2a*, whereas other PMNs do not. These findings raise the possibility that Islet2a plays a role in specifying CaP’s motor neuron subtype-specific properties.

In addition to *islet2a*, zebrafish embryos express *islet1* and *islet2b* [9, 10] [8] [13] [14]. All PMNs initially express *islet1* [8]. In zebrafish, knock-down of Islet1 results in failure of presumptive motor neurons to extend peripheral axons and innervate muscle targets, a fundamental requirement for motor function [15]. Further, upon disruption of Islet1 function, presumptive motor neurons differentiate novel non-motor neuron-like membrane electrical membrane properties [16]. These results implicate an essential role for Islet1 in general specification of motor neurons.

A few hours after the initial expression of *islet1*, CaPs begin to express *islet2a* and simultaneously downregulate *islet1* [8]. The exclusive expression of *islet2a* in CaP vs. other PMNs raises the possibility that it may selectively specify this motor neuron subtype. Consistent with this, disruption of Islet2a function, either by overexpression of Islet2a-LIM domains or morpholino (MO) knock-down, leads to defects in outgrowth of CaP axons [17] [15]. In addition, in *prdm14* mutants, CaPs lack expression of *islet2a* and have axons with stunted growth [18].

In *Drosophila* larvae, loss-of-function of *islet*, the fly orthologue of zebrafish *islet* genes, leads to changes in morphology as well as electrophysiological properties of one motor neuron subtype [19, 20]. CaP’s electrophysiological properties also distinguish it from MiP [12]. Specifically, the densities of inward and outward currents are 2.9 and 2.5-fold greater, respectively, in CaPs compared to MiPs. On this basis, we tested whether Islet2a determines CaP’s distinct electrophysiological as well as morphological properties. The results do not support a specific, non-redundant role for Islet2a in determining CaP’s stereotypic large inward and outward current densities.

## Materials and methods

### Zebrafish transgenic lines

All animal procedures used in this study have been approved by the University of Colorado Denver Animal Care and Use Committee (Approval number—74810(04)1D). Embryos and larvae were sacrificed by tricaine overdose accomplished by prolonged immersion in 0.02% tricaine methane sulfonate (Sigma-Aldrich, St. Louis, MO).

Several transgenic lines that express green fluorescent protein (gfp) in specific subpopulations of motor neurons were used for this study: tg(mnx1:gfp)ml2Tg [21]; tg(gata2:gfp)zf35Tg [22]; tg(islet1:gfp)rw0Tg [23, 24]. Here, we refer to these lines as tg(mnx1:gfp), tg(gata2:gfp) and tg(islet1:gfp), respectively. All motor neurons express gfp in the tg(mnx1:gfp) line, whereas dorsally- and ventrally- projecting secondary motor neurons (SMNs) express the reporter in the tg(gata2:gfp) and tg(islet1:gfp) lines, respectively.

### Gene-editing using transcription activator-like effector nucleases (TALEN) technology

Mojo Hand was used to design TALEN constructs that targeted the *islet2a* gene [25]. TALEN arms were limited to 15 repeat variable di-residues (RVDs) in length [26]. The TALEN pair, *islet2a*Tal1 (5’-CAG TAC CTG GAT GAG-3’), and *islet2a*Tal2 (5’-GTC CGA GAC GGC AAG-3’), targeted sequences in exon 2 of *islet2a* and flanked an ApaL1 restriction enzyme site (underlined) within a 15-base pair (bp) spacer (5’-ACGTGCACTTGCTTC -3’). The corresponding 15mers were: Tal1 RVD (HD NI NN NG NI HD HD NG NN NN NI NG NN NI NN) and Tal2 RVD (HD NG NG NN HD HD NN NG HD NG HD NN NN NI HD). TAL assembly was based on methods previously described [27]. Proper assembly of RVD-module vectors during the first Golden Gate reaction was confirmed by PCR, restriction digest, and DNA sequencing. Capped RNA was synthesized with the mMessage mMachine T3 transcription kit (Thermo Fisher Scientific, Waltham, MA). TALEN RNA pairs, 90–279 ng/arm, were injected into one-cell stage embryos. To test for TALEN induced recombination events, genomic DNA was extracted from single injected 24 hours post fertilization (hpf) embryos. PCR was then performed with *islet2a* primers: forward (5’-GATATTCGGGGTCCAGGTTT-3’) and reverse (5’-CGCTGCTTTTATCTCCAGTTT-3’) followed by ApaL1 test digests. Talen injected embryos were raised, and adults (G0) were outcrossed to wildtypes to identify founders. PCR and DNA sequencing analysis were performed on genomic DNA of F1 (heterozygote) and F2 (heterozygote and homozygote) embryos to obtain the exact recombination sequences (Barbara Davis, Sequencing Core, University of Colorado Anschutz Medical Campus).

**Fig 1:**
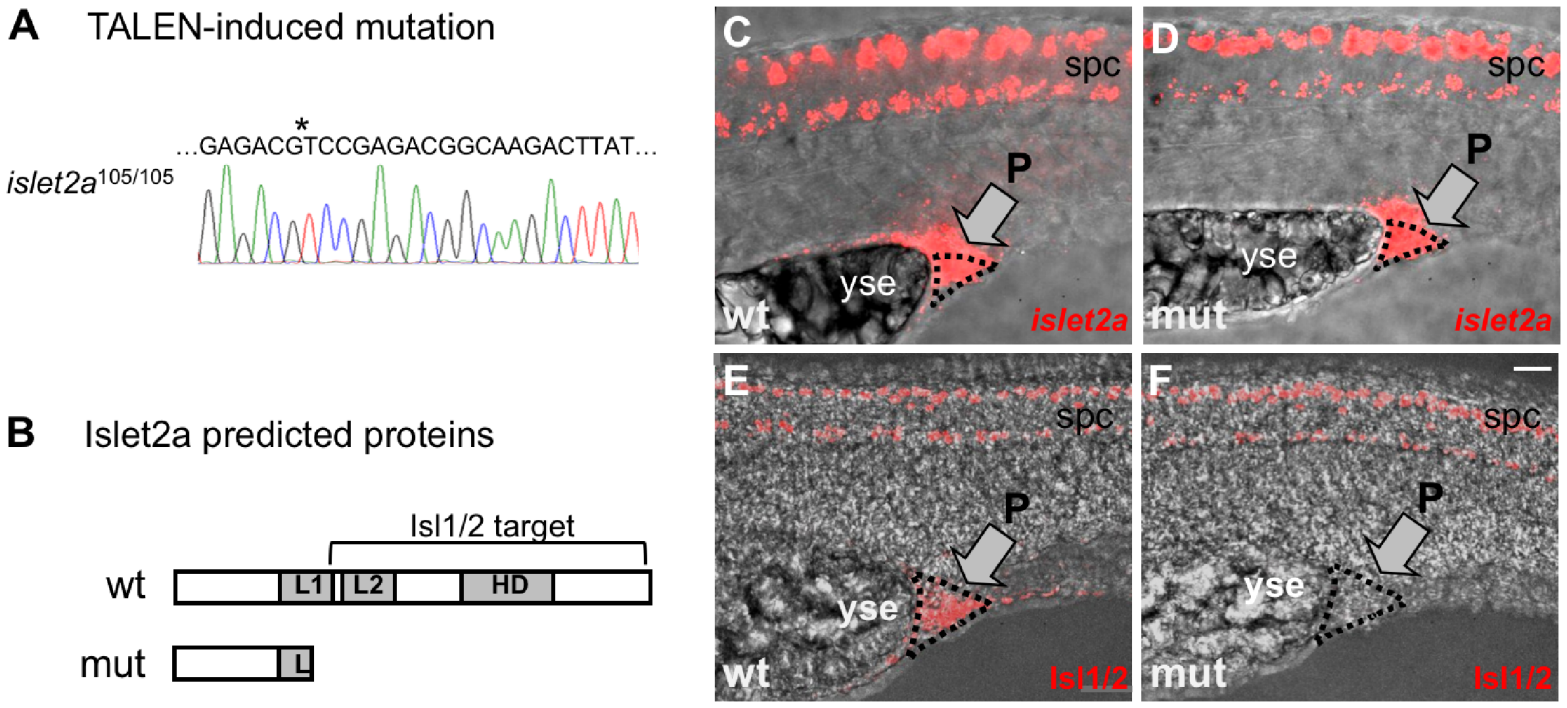
The *islet2a* TALEN mutant. (A) The sequence of homozygous *islet2a*^105^ genomic DNA revealed a 13-nuc deletion (at position *) that removed a restriction enzyme site and introduced a frameshift. (B) Schematic of wildtype and mutant Islet2a proteins. The *islet2a*^105^ sequence predicts a truncated Islet2a protein product lacking the homeobox (HD) and LIM2 (L2) domains and containing only a portion (~70%) of the LIM1 (L1) domain. The Isl11/2 monoclonal antibody used in this study was raised against a carboxy-terminus sequence not present in the predicted mutant protein. (C-F) Expression of *islet2a* mRNA (C, D) and Isl1/2 immunoreactivity (E, F) in wildtype (wt) and homozygous mutant (mut) *islet2a* 28 hpf embryos. Images are lateral views of embryos, with dorsal up and anterior to the left. Bright field and fluorescent images have been merged. (C, D) A tissue that expresses *islet2a*, but not *islet1* or *islet 2b*, was used to assess the efficacy of the TALEN mutation. The proctodeum (P), just caudal to the posterior end of the yolk sac extension (yse) emerges during embryonic stages and develops later into the anal passage. In both wildtype (n = 6) and mutant (n = 8) embryos, the proctodeum expressed *islet2a* mRNA. (E, F) The Isl1/2 antibody immunolabeled the proctodeum in wildtype (E; n = 10) but not mutant (F; n = 7) 28 hpf embryos. Scale bar in F, for C-F: 25 μm.

We isolated two different mutations, 105(CO7002) and 102(CO7003). Both had frameshifts introduced within exon 2 leading to a downstream premature STOP codon (Fig 1A) The 105 allele consisted of a 13-nucleotide deletion within exon 2, leading to a frame shift and premature STOP codon. The second allele, CO7003, also had a frameshift at the same position and a premature stop codon following 30 non-conserved amino acids. For both alleles, homozygous mutants were viable and to adult stages. Here, we report results for the 105 allele.

### Morpholino knockdown

Antisense MO oligonucleotides (Gene Tools, Philomath, OR) were injected into one-cell stage zebrafish embryos. We used a previously published translation blocking antisense, T-MO [15], directed to the start codon of the *islet2a* mRNA sequence (5’- GGATGCGGTAGAATATCCACCATAC-3’) at a concentration of 5 mg/ml. This T-MO differs from but partially overlaps with another T-MO previously used to perturb Islet2a function [17]. We used a control MO (Ctl MO) with 5-base pair mismatches compared to the T-MO (5’-GaATGCGcTAcAATATCCAgCAaAC-3’) at a concentration of 5 mg/ml. In addition, a splice blocking MO, Sp-MO (10 mg/ml) was designed to a sequence overlapping the splice junction between intron 1–2 and exon 2 (5’-CAGACTTCTCTGGATATGGAAAGCA-3’; S1 Fig). Comparisons of the MO and *islet* gene sequences supports specificity of the MOs targeting *islet2a* (Table 1 and S1 Table) [28, 29]. The T-MO and Sp-MO produced similar motor neuron morphological results and we report results obtained with T-MO.

**Table 1:** MO specificity for *islet2a*.

	Number of Mismatches with Target		
Gene	T-MO	Ctl MO	Sp-MO
***islet1***	0/25	5/25	0/25
***islet2a***	14/25	15/25	12/25
***islet2b***	9/25	10/25	8/25
***isl1l***	18/25	17/25	19/25

For *islet1* and *islet2b*, the regions corresponding to the *islet2a* sequences targeted by the MO were identified and compared. We include i*sl1l*, identified as the duplicate of *islet1* on the basis of syntenic relationships [28, 29]. However, zebrafish embryos express little to no *isl1l* mRNA (zfin.org). Further, the predicted Isl1l amino acid sequence shares only 47% identity with *islet1*. S1 Table presents the sequences for the regions of interest in the *islet* genes and the sequence of the intended MO targets, to show the distribution of the mismatches.

### RNA *in situ* hybridization

RNA *in situ* hybridization was carried out on fixed whole-mount 24, 28, 30 and 48 hpf embryos, as described previously [30]. Briefly, embryos were fixed in 4% paraformaldehyde, followed by dehydration with increasing concentrations of methanol and overnight incubation in 100% methanol (-20°C). Digoxigenin-labeled sense and antisense *islet1*, *islet2a* and *islet2b* RNA probes [8, 31] were synthesized and hybridized to whole-mount preparations [32, 33]. Hybridized RNA probes were detected with anti-digoxigenin Fab fragments coupled to alkaline phosphatase, followed by reaction with the Fast Red chromogen (Sigma-Aldrich, St. Louis, MO). In most cases, RNA *in situ* hybridization was followed by immunocytochemistry.

### Immunocytochemistry

For whole mount immunohistochemistry, 24–72 hpf embryos and larvae were fixed in 4% paraformaldehyde in PBS. Antibodies were diluted in 10% heat inactivated goat or fetal bovine serum in phosphate-buffered saline (PBS) containing 0.2% Tween. When immunochemistry followed *in situ* hybridization, 2% bovine serum albumin (Sigma-Aldrich, St. Louis, MO) was also included.

For detection of Islet protein, we used an antibody that recognizes both Islet1 and Islet2 proteins (Isl1/2 monoclonal; 39.4D5, Developmental Studies Hybridoma Bank [DSHB], Iowa City, IA; 1:500). CaP motor neurons were identified by immunolabeling using a combination of monoclonal zn1 (DSHB; 1:200) and monoclonal anti-syt2b (znp1; DSHB; 1:1000), or polyclonal anti-gfp (Thermo Fisher Scientific; 1:700–1000) when the tg(mnx1:gfp) line was used. To reveal SMN axons, we used the neurolin monoclonal, zn8 (1:200; DSHB). The following Alexa-conjugated secondary antibodies (Thermo Fisher Scientific) were used at 1:1000: goat anti-rabbit IgG Alexa-488; goat anti-mouse IgG_2b_ Alexa-546; goat anti-mouse IgG_2a_ Alexa-488; goat anti-mouse IgG_1_ Alexa-488 or -546.

The number of dorsally-projecting SMNs (dSMNs) were counted in 72 hpf tg(islet1:gfp) larvae in 4 hemisegments above the junction between the yolk sac and yolk sac extension. For purposes of comparison, we normalized values to the average number in uninjected larvae. Data were analyzed for statistical analysis by ANOVA followed by *post hoc* Bonferroni to correct for multiple comparisons.

### Confocal imaging

For imaging of processed embryos, we used a Marianas spinning disk confocal microscope (Intelligent Imaging Innovations, Denver, CO). Images were taken in the region comprising the ~3–4 hemisegments caudal and rostral to the junction between the yolk sac and yolk sac extension, except when examining the more-caudally located proctodeum. The same settings (e.g., gain, laser intensity) were used for imaging all samples in an experiment. Z-stack projections were made using open source software (FIJI version of Image J) [34, 35]. Sample size information is provided in the figure legends.

### Electrophysiology

Electrophysiology was performed as described previously [12, 16, 36]. Briefly, 24 or 48 hpf embryos were anesthetized with tricaine (0.02%, Sigma-Aldrich, St. Louis, MO) and secured to the bottom of a sylgard-lined recording chamber using suture glue (Vetbond, 3M, Maplewood, MN). Embryos were killed by hindbrain transection, skinned and rinsed with Ringer’s solution (in mM: 145 NaCl, 3 KCl, 1.8 CaCl_2_ and 10 HEPES, pH 7.4). Prior to recording, the Ringer’s solution was replaced with external solution (in mM: 125 NaCl, 2 KCl, 10 CaCl_2_ and 5 HEPES, pH 7.4). α-Bungarotoxin was used at ~0.8 μM to immobilize embryos during recordings. Voltage clamp recordings were obtained from spinal cord neurons that express *islet2a*: CaPs, ventrally-projecting secondary motor neurons (vSMNs) and Rohon-Beard cells (RBs) using an Axopatch 200B amplifier (Axon Instruments, Molecular Devices, Sunnyvale, CA). Electrodes for recordings (2.5 to 3 MOhms) were made from borosilicate capillary glass (Drummond Scientific, Broomall, PA) using a P-97 microelectrode puller (Sutter Instruments, Novato, CA).

The holding potential was set to -80 mV and trials consisting of 16 50 msec depolarizing steps; each successive step varied by +10 mV starting at -40 mV and extending to 110 mV. For each step, before returning the membrane potential to -80 mV, a 20 msec step to -40 mV was applied to record tail currents. For presentation of data, exemplar traces for steps to +20 mV are shown.

For calculation of current density, current amplitudes were normalized to cell size by dividing by the cell capacitance, a measure of the cell’s surface area. For inward and outward current density calculations, we used the peak inward current amplitude recorded during the entire trial of 16 steps and the steady-state outward current recorded during the final 20 msec of the step to +40 mV, respectively. Sample size information is provided in the figure legends.

### Whole embryo RNA isolation

RNA was isolated from 30–60 pooled 48 hpf whole embryos of the following conditions: *islet2a* sibling wildtype, *islet2a* mutant; sibling wt uninjected, wildtype injected with T-MO or Ctl MO. For microarray analyses, wildtype and uninjected embryo RNAs were each collected in duplicate, and RNA of mutants and morphants in triplicate. For quantitative polymerase chain reaction (qPCR) studies, all samples were collected in triplicate. RNA was isolated by column purification using the RNeasy Mini Kit (Qiagen, Venlo, Netherlands). RNA concentration and integrity were assessed using an Agilent 4200 Tape Station (Agilent Technologies, Santa Clara, CA).

### Microarray analysis of gene expression

RNA was delivered to the University of Colorado Denver Genomics and Microarray Core for analysis of gene expression using RNA microarrays (GeneChip^™^ Zebrafish Gene 1.0 ST Array, Thermo Fisher Scientific). A hybridization cocktail was prepared using 100 ng total RNA and the GeneChip WT PLUS Reagent Kit (Thermo Fisher Scientific). Samples were hybridized to the arrays for 16 hrs at 45°C (GeneChip^™^ Hybridization Oven 645, Thermo Fisher Scientific). Arrays were washed, stained (GeneChip^™^ Fluidics Station 450, Thermo Fisher Scientific), and scanned using a GeneChip Scanner 3000 (Thermo Fisher Scientific). Expression data from the resulting CEL files (see Supplementary information) were extracted and the OLIGO package in R was used to perform RMA (robust multichip average) normalization (S1 File). From this, pairwise ANOVAs in R were performed to compare the morphant vs. mutant, mutant vs. control and morph vs. control datasets; the two wildtype and two uninjected wildtype samples comprised the control group.

Two criteria were used to identify differentially expressed (DE) genes: (1) Q (p corrected for a false discovery rate of 5%) <0.05; (2) fold expression change that was either <-2 or >2. Heat maps were constructed using online software (https://software.broadinstitute.org/morpheus). We used Ingenuity Pathway Analysis^®^ (IPA^®^, Qiagen, Venlo, Netherlands) to identify pathways that were potentially differentially modified.

### cDNA synthesis and quantitative polymerase chain reaction

RNA samples were treated with Amplification Grade DNase (Thermo Fisher Scientific) prior to synthesis of cDNA. cDNA synthesis and qPCR were performed using the EXPRESS One-Step Superscript qRT-PCR Kit and a 7500 Fast Instrument (Thermo Fisher Scientific). Table 2 provides the assay IDs for the genes studied: *islet1*, *islet2a*, *islet2b*, *nrp1a*, and *plexinA3*. The assays targeted a region that flanked an intron, to allow distinction between amplification of mRNA vs. genomic DNA. Gene expression levels were normalized to that of the housekeeping gene *eef1a1a*.

**Table 2:** qPCR gene assays.

Target	Thermo Fisher Scientific Assay ID	Exons Targeted
*islet1*	Dr03425734_m1	4–5
*islet2a*	Dr03124888_m1	4–5
*islet2b*	Dr03111925_m1	5–6
*nrp1a*	Dr03106127_m1	11–12
*plexinA3*	Dr03149727_m1	21–22
*eef1a1a*	Dr03119741_m1	1–2

Cycling conditions were adjusted to those specified by the kit manufacturer (Thermo Fisher Scientific). The biological triplicates were each run in technical triplicate. The data were analyzed with the ABI 7500 Software Version 2.0.6 using all default parameters except that the threshold for the C_t_ standard deviation was changed from >0.5 to >0.3. The relative standard curve method was used with gene expression normalized to that of *eef1a1a*. The validity of *eef1a1a* as the endogenous control was tested by measuring the standard deviation of the threshold cycle (C_t_) of all the samples of equal concentrations. The standard deviation was consistently below 0.5, indicating that *eef1a1a* did not vary significantly between samples.

For analysis of statistical significance, the three technical replicates for each biological replicate were first averaged. Then, the means of the biological triplicates for each condition (wildtype, *islet2a* homozygous mutants, embryos injected with T-MO or Ctl MO) were calculated; sibling wildtype and uninjected wildtype were pooled as one group, referred to as wildtype. Statistical comparisons were done using ANOVA followed by *post hoc* Bonferroni to correct for multiple comparisons.

## Results

### Disruption of the *islet2a* gene

Using TALEN based methods, we introduced a 13-nucleotide deletion into exon 2 of *islet2a*, leading to a frame shift and premature STOP codon (105 allele; Fig 1A). The predicted truncated protein lacks the majority of the LIM1 domain and all of the LIM2 and homeobox DNA-binding domains (Fig 1B), regions required for function as well as recognition by the Isl1/2 antibody.

Many cells coexpress *islet2a* with other *islet1* genes [14, 31]. A notable exception is the proctodeum that expresses *islet2a* but not *islet1* [18]. RNA *in situ* hybridization demonstrated that both wildtype and mutant embryos expressed *islet2a* mRNA at comparable levels in the proctodeum as well as the spinal cord (Fig 1C and 1D). This result indicates that the TALEN-induced mutation in exon 2 of *islet2a* did not trigger nonsense mediated decay, a mechanism that targets and degrades many, but not all, mRNAs containing premature stop codons [37].

Analysis of Islet protein expression in zebrafish tissues typically involves use of a monoclonal antibody, Isl1/2, that recognizes both Islet1 and Islet2 proteins. The proctodeum of wildtype embryos showed positive Isl1/2 immunoreactivity as did cells within the spinal cord (Fig 1E), consistent with *islet2a* expression in the proctodeum. In *islet2a* mutants, however, the proctodeum lacked Isl1/2 immunoreactivity (Fig 1F), as predicted upon loss of full-length wildtype Islet2a protein. These data indicate that the TALEN-induced mutation of *islet2a* led to loss of expression of full-length Islet2a protein.

In addition, we used a previously reported translation blocking antisense MO (T-MO) to investigate the role of *islet2a* in the zebrafish embryo [15]. As controls for the T-MO, we used a MO (Ctl MO) with 5 mismatches compared to T-MO and a splice-blocking MO (Sp-MO) that targets the junction between intron 1–2 and exon 2 of the *islet2a* gene (S1 Fig). For the Sp-MO, RT-PCR analysis revealed the presence of two novel bands (S1B Fig). The additional transcripts corresponded to forms predicted by retention of intron 1–2 (S1A Fig, a) or skipping of exon 2 (S1A Fig, b), as confirmed by DNA sequencing, as expected upon targeting of the junction between intron 1–2 and exon 2 by the Sp-MO.

#### Effects of gene-editing and MO targeting of *islet2a* on spinal neuron electrical membrane properties

Our previous work has shown that the electrical membrane properties of CaP differ significantly from another PMN: in 24 hpf embryos, compared to MiPs, CaPs had 2–3 fold larger densities of both inward and outward currents [12]. If the unique expression of *islet2a* in CaPs determines its stereotypic larger inward and outward current densities, loss of Islet2a would be expected to result in obvious decreases in CaP current densities.

**Fig 2:**
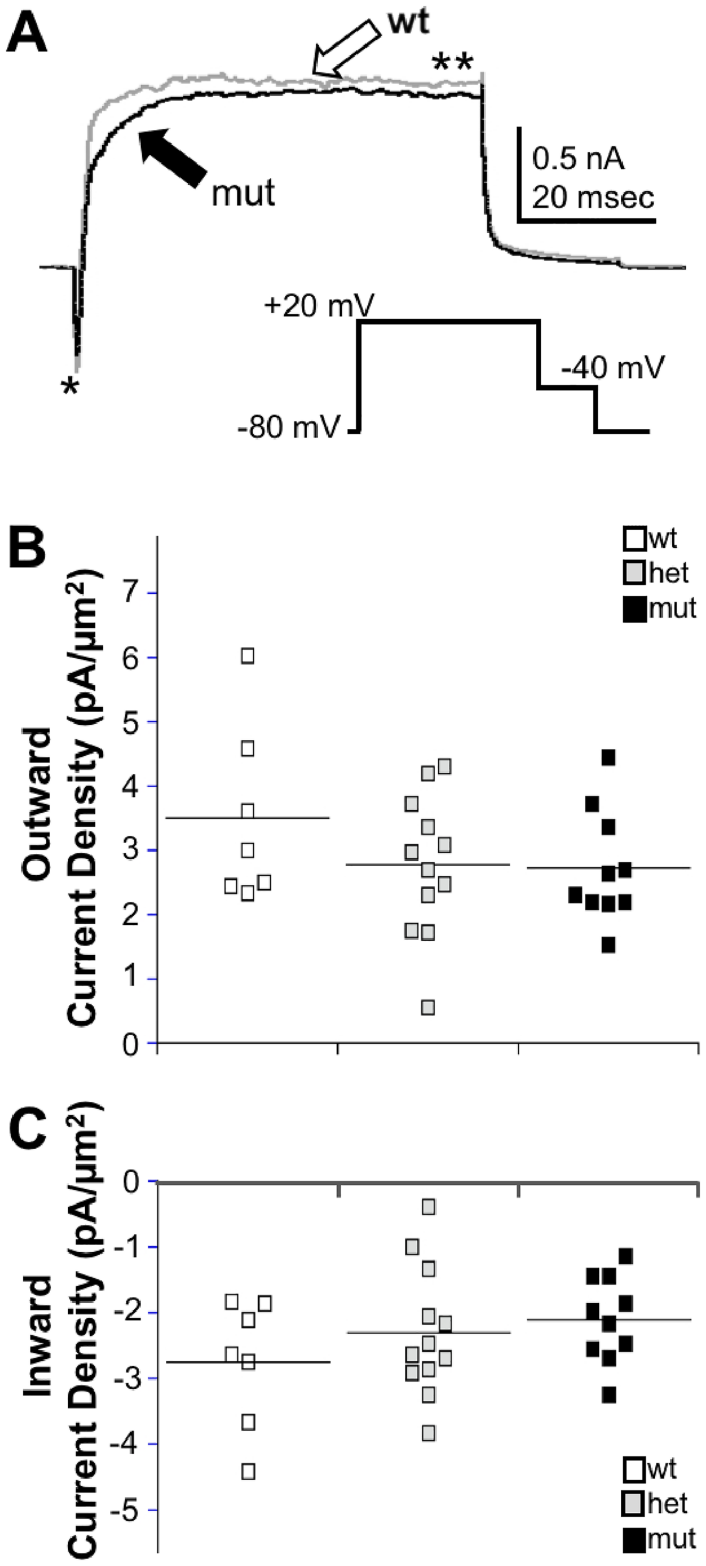
CaP current densities do not differ significantly in islet2a mutants compared to wild type or heterozygous embryos. (A) Recordings were obtained from CaPs in 24 hpf sibling (grey trace) and mutant (black trace) *islet2a* embryos. No obvious differences were noted in the amplitudes of peak inward (single asterisk) or outward (double asterisk) currents between conditions. The inset shows the voltage protocol used to elicit current. (B) The scatter plot presents the values of outward current densities for each CaP recorded from in wildtypes (wt; n = 7, 5 embryos), heterozygotes (het; n = 12, 9 embryos), and mutants (mut; n = 10, 6 embryos). The horizontal lines denote the mean value for each group (ANOVA; p values: ctl vs. het, 0.50, wt vs. mut, 0.49; het vs. mut, 1). (C) Similar to outward current densities, peak inward current densities did not differ significantly between CaPs recorded from in wildtype, heterozygous, and mutant embryos (ANOVA; p values: ctl vs. het, 0.83, wt vs. mut, 0.41; het vs. mut, 1).

In 24 hpf tg(mnx1:gfp) embryos, CaPs were identified on the basis of gfp expression, soma position and a ventrally-projecting axon. We obtained whole cell voltage clamp recordings from CaPs in 24 hpf *islet2a* mutant and sibling wildtype embryos (Fig 2A). In contrast to our prediction, we did not detect statistically significant differences in either inward or outward densities for data obtained from CaPs in *islet2a* wild type, heterozygous or homozygous mutant embryos (Fig 2B and 2C). However, even though the mean values of outward current densities were not significantly different statistically, there was a small difference in the means of CaP outward current density recorded from wildtype compared to mutant embryos (wildtype: 3.5±0.5 pA/μm^2^; heterozygote: 2.8±0.3 pA/μm^2^; mutant: 2.7±0.3 pA/μm^2^). It is possible that with a larger sample size, we might have detected a statistically significant difference between wildtype and mutants. However, on the basis of our results, the difference would not fully account for the previously reported ~3-fold difference in outward current densities of CaPs compared to MiPs [12].

**Fig 3:**
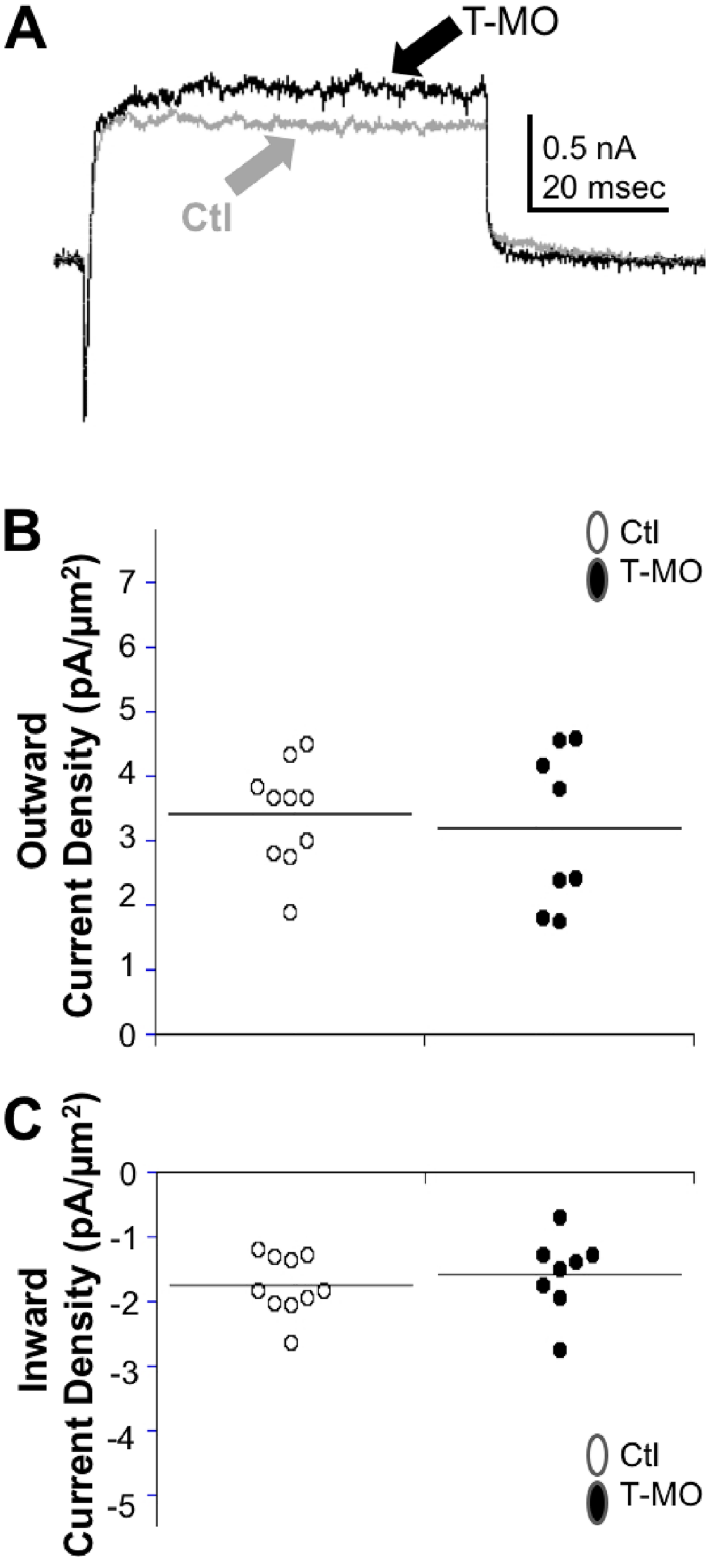
MOs targeting Islet2a function do not produce significant effects on CaP whole cell voltage-dependent currents. (A) Recordings were obtained from CaP neurons in 24 hpf uninjected control (grey trace) and morphant (black trace) *islet2a* embryos. No obvious differences were noted in the amplitudes of peak inward or outward currents. The voltage protocol is as in Fig 2A. (B) The individual values of outward current densities each CaP recorded from in controls (n = 10 cells, 6 embryos) and morphants (n = 8 cells, 8 embryos) are shown. The values for the two groups did not differ significantly (unpaired two-tailed Student t-test: p value, 0.65). (C) Similar to outward current densities, the densities of peak inward currents recorded from CaPs in the control and morphant embryos were not statistically different (unpaired two-tailed Student t-test: p value, 0.51).

Whole cell voltage clamp recordings were also obtained from *islet2a* morphant and control embryos. Similarly, no significant differences were noted between CaPs in morphant and control embryos for either inward or outward current densities (Fig 3).

Other spinal neurons, e.g., vSMN and RB, also express *islet2a* [8, 14, 31] (S2 Fig) leading us to test whether disruption of Islet2a altered electrical membrane properties of these cells. Similar to CaPs, vSMN inward and outward currents showed no obvious differences in recordings obtained from uninjected vs. morphant 48 hpf embryos (S2A and S2B Fig). We also recorded from RBs, given the widespread expression *islet2a* in this population [31]. However, we detected no significant differences in the densities of inward or outward current of RBs in uninjected vs. morphant embryos, at either 24 or 48 hpf (S2C and S2D Fig).

Overall, these findings do not support a unique role for Islet2a in specifying electrophysiological properties of vSMNs or RBs. However, *islet2a* is not expressed in all vSMNs (S2 Fig). In addition, RBs co-express several *islet* genes [14, 31] and Islet1 can substitute for Islet2a [15]. Thus, we cannot rule out the possibility that Islet2a normally plays a role in differentiation of RB’s electrical membrane properties but in its absence, other Islet proteins effectively compensate. In contrast, at 24 hpf, only *islet2a* is detected in CaPs, making the possibility of compensation by another normally co-expressed *islet* gene unlikely.

### Effects of MO knock-down vs. Talen mutagenesis of *islet2a* on CaP morphology

**Fig 4:**
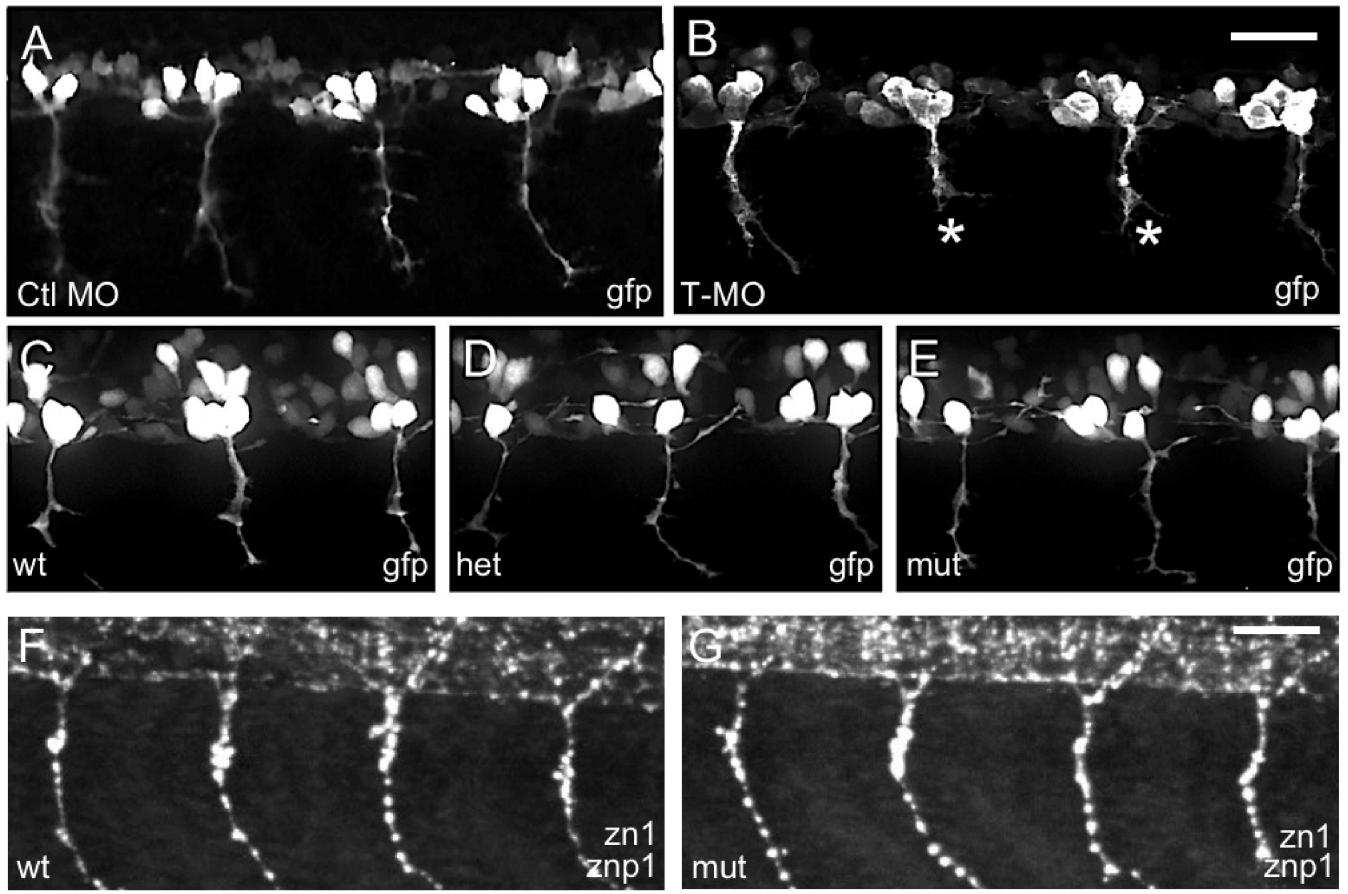
Islet2a morphant but not mutant embryos displayed altered CaP morphology. (A-E) In live *tg(mnx1*:*gfp)* 24 hpf embryos, CaP neurons expressed gfp in their somas and axons. (A, B) Injection of a T-MO (MO) led to truncation of ventrally projecting axons (asterisks, B) compared to control (Ctl, A), as previously reported [15, 17]. (C-E) In wildtype (wt, C), heterozygous (het, D) and mutant (mut, E) *islet2a* embryos, CaP neuron axon growth and trajectories appeared normal regardless of genotype. Sample size ranged from 8–30 per condition. Scale bar in E, for A-E: 50 μm. (F, G) In fixed non-transgenic 28 hpf embryos, zn1/znp1 immunoreactivity did not reveal any differences in CaP axon morphology between wildtype (F; n = 9) versus mutant (G; n = 9) embryos.

While performing the electrophysiological experiments, we observed that CaPs in morphants often had abnormal, truncated axons (Fig 4A and 4B), as reported previously when MOs or dominant-negative overexpression was used to perturb Islet2a function [15, 17]. In contrast, we did not observe truncated axons in live *islet2a* wildtype, heterozygous or mutant embryos (Fig 4C–4E). In mutants, CaP axons appeared normal on the basis of gfp expression in live *tg(mnx1*:*gfp)* 24 hpf embryos (Fig 4C–4E) or immunolabeling with axonal markers in fixed embryos non-transgenic (Fig 4F and 4G).

**Fig 5:**
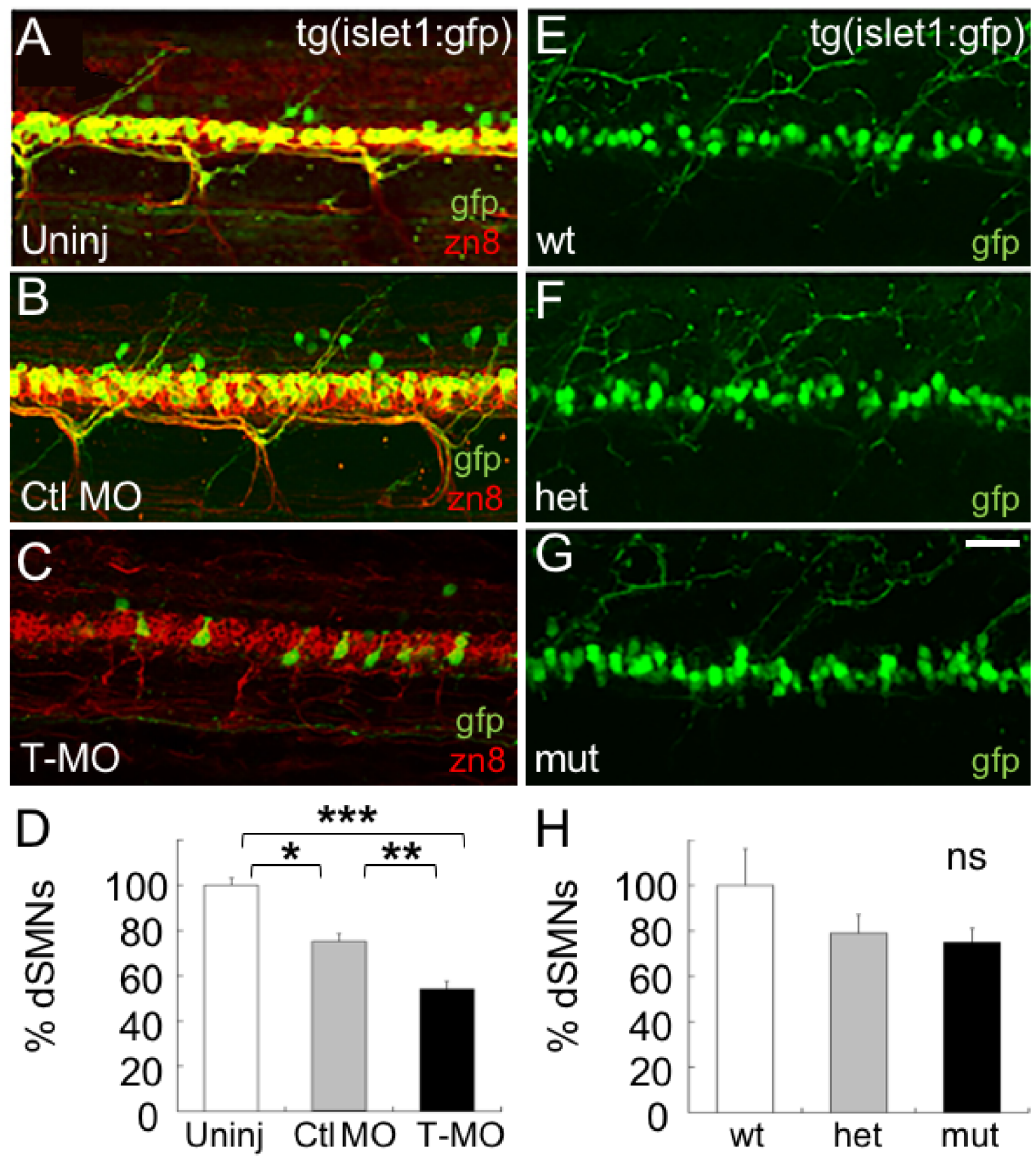
Islet2a morphant, but not mutant, larvae have a reduced number of dSMNs. (A-C) In 72 hpf tg(islet1:gfp) larvae, dSMNs expressed gfp in their somas and axons. In addition, the zn8 antibody recognized the neurolin protein (red), expressed on SMN somas and axons [38]. (A) In uninjected 72 hpf larvae, the majority of zn8^+^ neurons also expressed gfp. (B) Following injection of the control MO (Ctl MO), zn8^+^ (red) neurons continued to express gfp. In addition, dSMNs developed normally with respect to axon morphology (arrowhead), as assessed by zn8 immunolabeling (red). (C) Injection of the T-MO led to a decrease in the number of zn8^+^ neurons that coexpressed gfp. Scale bar in A, for A-C: 50 μm. (D) In tg(islet1:gfp) 72 hpf larvae, the numbers of gfp^+^ somas were reduced by injection of the Ctl MO (n = 17) and further reduced by injection of the T-MO (n = 20) compared to uninjected embryos (n = 10). ***, p<0.0001; **, p<0.0003; 8, p<0.0006; ANOVA with post-hoc Bonferroni. (E-G) In live 72 hpf tg(islet1:gfp) larvae, dSMNs appeared normal in number and morphology in homozygous mutant (mut; G) compared to heterozygous (het; F) and homozygous wildtype (wt; E) 72 hpf embryos. (H) Cell counts indicated that the number of dSMN somas was not reduced in mutant (n = 9) compared to wildtype (n = 5) or heterozygous (n = 8) *islet2a* 72 hpf embryos.

Islet2a morphants have also been previously observed to have a loss of dorsally-projecting SMNs (dSMNs) [15]. We examined dSMN axons in tg(islet1:gfp) 3 dpf larvae on the basis of gfp expression as well as zn8 (neurolin) immunoreactivity (Fig 5A–5C). As found previously, injection of the T-MO resulted in fewer dSMN axons in the periphery (Fig 5C vs. Fig 5A and 5B). In addition, the number of dSMN cell bodies was also reduced in morphant larvae (Fig 5D).

We then examined dSMN morphology in *islet2a* siblings and mutants. In contrast to morphants, we did not detect any dSMN phenotypes in *islet2a* siblings or mutants (Fig 5E–5H). In 72 hpf wildtype (Fig 5E), heterozygous (Fig 5F) and mutant (Fig 5G) tg(islet1:gfp) larvae, dSMNs appeared normal with respect to axonal morphology. Whereas heterozygotes and mutants showed a small reduction in the number of dSMN cell bodies, the difference was not statistically significant compared to control but statistically non-significant reduction in the number of dSMN cell bodies (Fig 5H). Overall, *islet2a* mutants had neither the CaP axon nor the dSMN phenotypes that were present upon injection of the T-MO (Figs 4 and 5).

### Comparison of gene expression in morphant and mutant embryos

The different motor neuron phenotypes of *islet2a* mutants vs. morphants raised questions about the extent to which mutants and morphants differed. Accordingly, we compared their transcriptomes using microarrays. For these analyses, we chose a single developmental stage, 48 hpf, that was intermediate between the two stages at which we observed differences in motor neuron morphologies between mutants and morphants.

**Fig 6:**
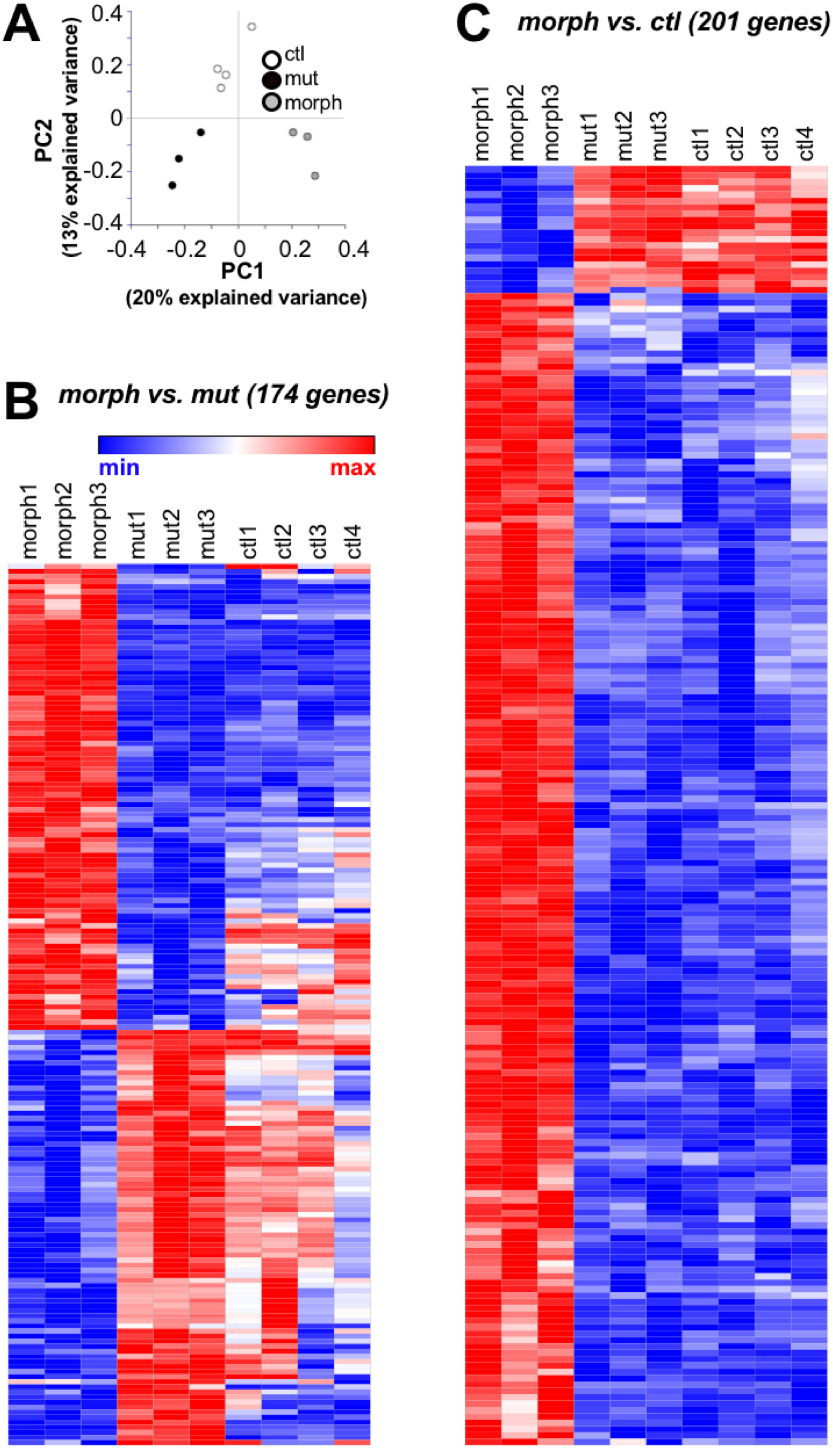
Microarray analysis revealed differential expression of gene in morphants compared to mutants or controls. (A) PCA analysis of gene expression profiles of 48 hpf control (ctl), mutant (mut) and morphant (morph) transcriptomes indicated that they sort into three different groups on the basis of the first two principal gene expression components (PC1, PC2). (B, C) Heat map plots of differentially expressed genes. Each row indicates one of the 174 genes. Expression is normalized by row: blue, red and white indicate minimum, maximum and midpoint expression levels, respectively. The data have been hierarchically clustered by rows (genes). (B) 174 genes were differentially expressed in mutant compared to morphant transcriptomes. The heat map also included control gene expression levels for purposes of comparison. Many of the 174 genes had expression altered in opposing directions in mutants and morphants compared to controls. (C) 201 genes were differentially expressed in morphants compared to controls. For purposes of comparison, the heat map also included mutant gene expression levels.

Principal component analysis of gene expression in control, mutant and morphant transcriptomes segregated them into different quadrants (Fig 6A). This provided an initial indication that the gene expression profiles of mutant and morphant transcriptomes differed from each other.

We identified 174 genes that were differentially expressed in morphants compared to mutants (Q<0.05; fold change <-2 or >2; S2 File). Including control values, a heat map revealed that these 174 genes were often regulated in opposing manners in morphants and mutants compared to controls (Fig 6B; S3 File). Compared to control, genes that were upregulated in the morphant (red) were often downregulated in the mutant transcriptomes (blue or less white) and *vice versa*.

Mutants did not have any genes that were differentially expressed compared to controls. In contrast, we identified 201 differentially expressed genes that were differentially expressed in morphants compared to control (Supplementary file S4 File). The heat map of expression levels of the 201 genes shows that gene expression in morphants and mutants, compared to controls, was often regulated opposing directions (Fig 6C; S5 File). There). Overall, there were 64 genes that were differentially expressed in morphants compared to both mutants and controls (S6 File). These comparisons further support the conclusion that morphant and mutant transcriptomes differed from each other.

Neither pathway analysis (IPA^®^) nor the identities of differentially expressed genes in morphants compared to mutants, controls or both provided any strong insights into potential mechanisms underlying the different motor neuron morphologies of mutants vs. morphants. For example, neither *nrp1a* nor *plexinA3* were identified as differentially expressed genes, even though they have been implicated in regulation of CaP axon morphology [39] [40]. To confirm the microarray results, we carried out focused analysis using qPCR and found that neither *nrp1a* nor *plexinA3* showed any differences in expression levels between wildtype, mutant and morphants embryos (S4 Fig).

**Fig 7:**
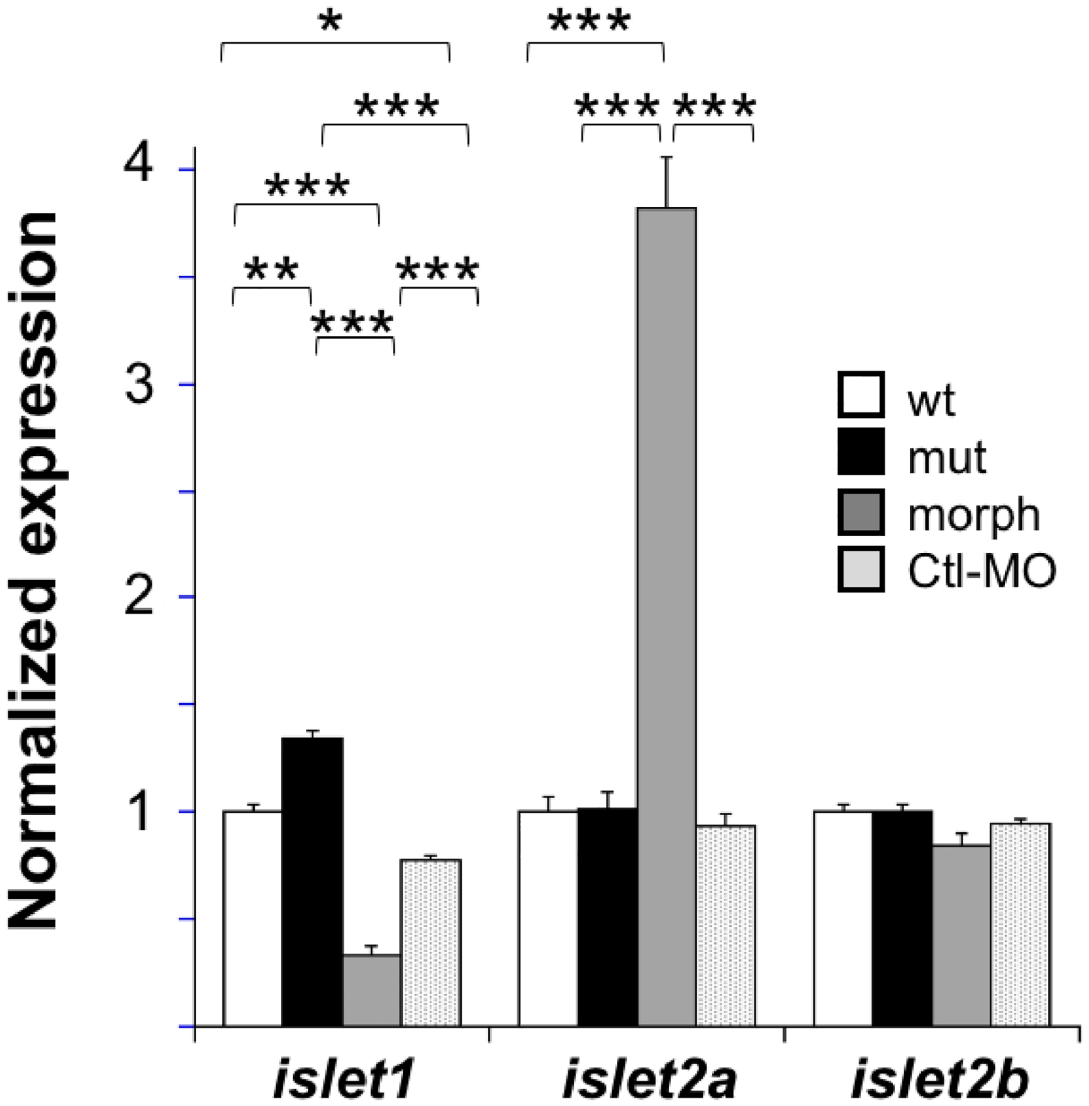
Relative levels of *islet* and downstream gene transcripts in morphants vs. mutants. qPCR was used to compare expression levels of *islet1*, *islet2a* and *islet2b* between wildtype (wt), mutant (mut), morphant (morph) and Ctl MO-injected (Ctl MO) embryos. In *islet2a* mutants, *islet1* levels were significantly increased by ~ 25%; no changes in the levels of other *islet* transcripts were detected in mutants. Both morphants and Ctl MO-injected embryos had significantly decreased levels of *islet1*. The largest change detected was a ~ 4-fold increase in *islet2a* levels in morphant compared to wildtype or mutant. ***, p<0.001; **, p<0.003; *, p<0.005; ANOVA with post hoc Bonferroni. To facilitate comparisons, for each gene, expression levels were normalized to that of the control group.

One gene that was differentially expressed between morphants and controls was *islet2a* (ENSDART0000012862). Interestingly, *islet2a* levels were upregulated in morphants. This led us to question whether other *islet* genes had different expression levels in morphants and/or mutants compared to controls. For this focused analysis, we performed qPCR for *islet1*, *islet2a* and *islet2b* using RNA isolated from wildtype, mutant, morphant, and Ctl MO-injected embryos. As predicted by the microarray results, the level of *islet2a* in morphants was increased almost 4-fold compared to either wildtype or morphants (Fig 7).

qPCR analyses also detected significantly different levels of *islet1* in mutant and morphant vs. control transcriptomes (Fig 7). Compared to control, *islet1* expression was increased slightly (~25%) in mutants but decreased to ~67% and 33% of the wildtype level in Ctl MO-injected and morphant embryos, respectively (Fig 7). We did not detect any changes in expression levels of *islet2b* between any of the conditions.

In summary, the microarray results support the view that mutants and morphants differed not only in motor neuron morphology but also in gene expression profiles. Further, focused examination of *islet* gene expression using qPCR revealed that MOs targeting *islet2a* and gene-editing of *islet2a* led to different changes in expression of *islet* genes.

### *islet* expression in CaPs of *islet2a* wildtype and mutant embryos

**Fig 8:**
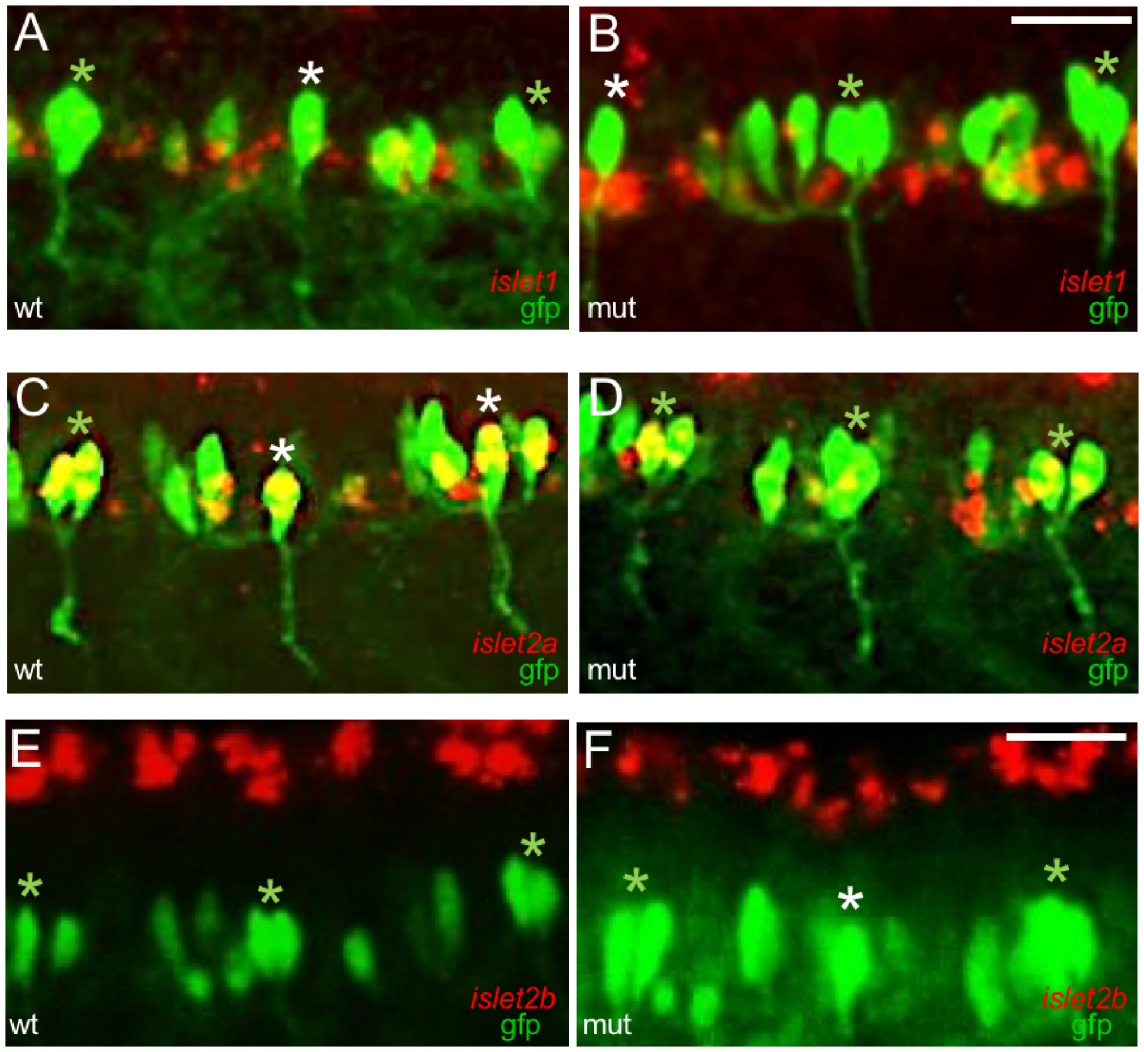
CaPs expressed *islet2a*, but not *islet1* or *islet2b*, in wildtypes and mutants. The expression patterns of *islet1* (A [n = 6], B [n = 8], *islet2a* (C [n = 6], D [n = 8]) and *islet2b* (E [n = 9], F [n = 8]) in CaPs were examined in 28 hpf sibling wildtype (A, C, E) and *islet2a* mutant (B, D, F) tg(mnx1:gfp) embryos. At 28 hpf, VaP (variably-present CaP duplicate) is present in some segments. In A-F, white and green asterisks denote lone CaPs and CaP/VaP pairs, respectively. Novel, ectopic expression of either *islet1* or *islet2b* in CaPs was not detected.

The microarray and qPCR analyses revealed changes in *islet* gene expression in 48 hpf whole embryo RNA. However, our electrophysiological studies focused on CaPs in 24–28 hpf embryos. To identify potential changes in *islet* gene expression that might have occurred specifically in 24–28 hpf CaPs, we preformed RNA *in situ* hybridization for *islet1*, *islet2a* and *islet2b*. In contrast to the microarray and qPCR analyses, RNA *in situ* hybridization did not detect any obvious changes in expression of any *islet* gene in CaPs of wildtype vs. mutant embryos (Fig 8). However, the change in *islet1* expression detected by qPCR was <2-fold (Fig 7), and RNA *in situ* hybridization is not a quantitative method. Further, as mentioned, RNA *in situ* hybridization focused on CaPs in 24–28 hpf embryos while the microarray and qPCR studies used RNA isolated from whole 48 hpf embryos. Overall, for CaPs, the RNA *in situ* hybridization results did not reveal any changes in *islet2a* expression or novel, ectopic expression of either *islet1* or *islet2b*.

### In *islet2a* mutants, Islet protein immunoreactivity persists in CaPs

Previous work demonstrated that CaPs in 28 hpf *islet2a* morphants lacked Isl1/2 immunoreactivity ([15]-Fig 6D therein). In contrast, 28 hpf *prdm14* mutants, that lack detectable *islet2a* in CaPs, had reduced Isl1/2 immunolabeling in these PMNs ((18)-Fig 6C therein). As the Isl1/2 antibody recognizes the carboxy-terminal regions of Islet1 and Islet2, the reason for persistence of CaP Isl1/2 immunolabeling in *prdm14* mutants is not obvious. Given these previous results, we examined Isl1/2 immunolabeling in CaPs of *islet2a* mutants.

**Fig 9:**
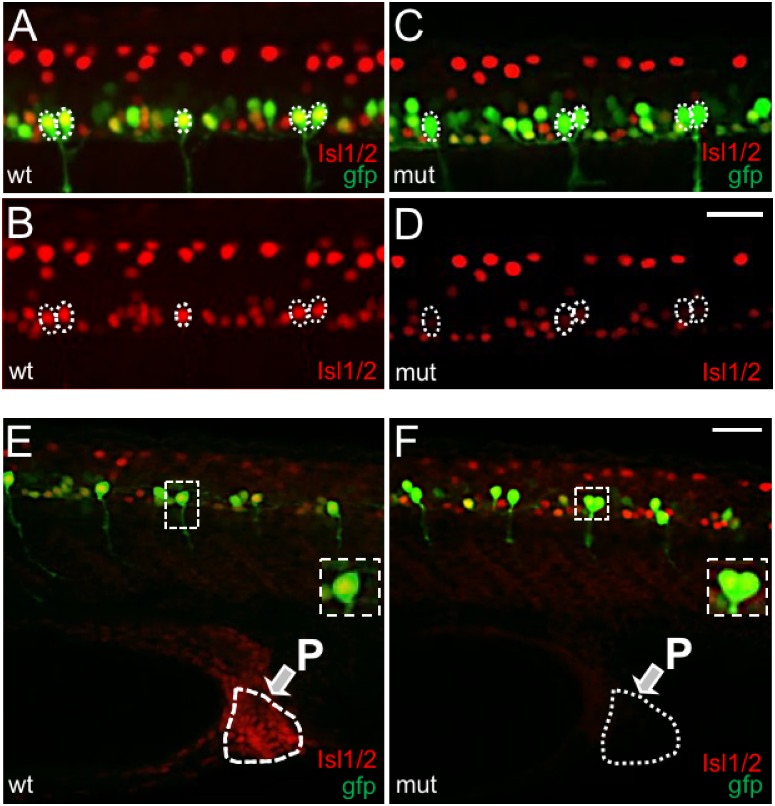
CaPs expressed reduced levels of Isl1/2 immunoreactivity in *islet2a* mutant embryos. (A) In 28 hpf tg(mnx1:gfp) wildtype (wt, n = 15) embryos, CaPs (*) expressed both gfp (green) and Isl1/2 immunoreactivity (red), as revealed by the merged yellow signal. (B) The Isl11/2 immunosignal of Panel A is shown separately. In A-D, dotted lines circle the cell bodies of CaP/VaPs that were immunopositive for Isl1/2. In comparison to other ventral neurons, CaP Isl1/2 immunolabeling was more intense. (C) In 28 hpf tg(mnx1:gfp) mutant (mut, n = 14) embryos, CaPs (*) expressed gfp. However, compared to wildtype (A), the CaP Isl1/2 fluorescent immunolabel signal was less intense. Further, other ventral neurons continued to display Isl1/2 immunoreactivity. (D) The Isl1/2 signal of Panel C is viewed separately. Compared to wildtype (B), CaPs expressed reduced levels of Isl1/2 immunoreactivity. Further, despite the weak signal in CaPs, Isl1/2 immunoreactivity was present in other ventral neurons at levels similar to wildtype (B). Scale bar in D for A-D: 25 μm. (E, F) Examination of CaP Isl1/2 immunolabeling dorsal to the proctodeum. (E) In wildtype embryos, both the proctodeum (white arrow) and CaPs (asterisks) displayed Isl1/2 immunolabeling. One CaP, contained within white dotted line box, is shown at higher magnification in the inset. (F) In mutant embryos, Isl1/2 immunolabeling was not detected in the proctodeum, consistent with loss of Islet2a protein expression. Despite this, a low level of Isl1/2 immunoreactivity persisted in CaPs (asterisks; one CaP shown at higher power in inset).

Similar to *prdm14* mutants [18], CaPs in *islet2a* mutants were positive for Isl1/2 immunolabeling at a reduced level compared to wildtypes (Fig 9A, 9B and 9E vs. Fig 9C, 9D and 9F). This result differs from what has been reported for *islet2a* morphants [15]. While inefficient knock-out of Islet2a protein in the mutant is a potential possibility in principle, we did not detect any Isl1/2 immunolabeling in the proctodeum, a tissue that expresses *islet2a* but not *islet1* (Fig 1E vs. Fig 1F). Despite the lack of novel expression of *islet1* or *islet2b* in mutant CaPs (Fig 8), the results suggest that a non-Islet2a Islet protein was present in CaPs of 28 hpf mutants.

## Discussion

Motor neuron subtypes differ not only with respect to peripheral axon trajectories and muscle targets but also electrical membrane properties [12, 20]. However, little is known about the mechanisms that direct differentiation of vertebrate motor neuron subtype-specific electrical membrane properties. In contrast, several studies implicate LIM-HD transcription factors in specification of motor neuron subtype-specific morphological properties (for review, [2, 3, 41, 42]. In *Drosophila*, *islet* (orthologous to zebrafish *islet1* genes) is expressed in ventral, but not dorsal, motor neurons [19]. Further, loss of *islet* results in loss of dorsal motor neuron’s distinguishing axonal morphology and larger outward current densities [20, 43–45]. These studies suggest that the same transcription factor code might specify motor neuron subtype-specific differentiation of electrical membrane properties as well as morphological differentiation, a possibility not yet tested in vertebrates.

Prior studies have tested for effects of Islet2a functional disruption on specification of CaP axon morphology [15, 17]. Overexpression of a dominant-negative Islet2a construct, consisting of only the Islet2a LIM domains, led to disruption of CaP axon morphology [17]. Even though the LIM domains are conserved across the different Islet proteins, the effect could be rescued by overexpression of Islet2a but not Islet1or Islet2b, suggesting a specific requirement for Islet2a. These authors also showed that an *islet2a* T-MO had similar but less severe effects on CaP axon morphology. Similarly, Hutchinson and Eisen [15] found that a different *islet2a* T-MO (the same one used here) impaired CaP axon morphology to a lesser extent than did overexpression of dominant-negative Isl2 constructs [17]. However, in contrast to the results of Segawa et al. [17], Hutchinson and Eisen [15] rescued effects of the *islet2a* T-MO on CaP axon morphology by overexpression of either *islet1* or *islet2a* mRNA. Murine *islet2* and *hb9* mutants both have a decreased number of one the the visceral motor neuron subtype, the visceral motor neuron [46–48]. Given that *islet2* and *hb9* mutants have in common a reduction in *islet1* levels, Thaler et al. [47] proposed that the requirement for Islet2 reflects a need for a specific total concentration of all Islet proteins, regardless of identity as Islet1 or Islet2. Thus, Segawa et al.’s [17] findings support a specific non-redundant role for *islet2a* in differentiation of motor neuron subtype-specific properties, while those of Hutchinson and Eisen [15] and Thaler et al. [47] do not.

Here, we tested whether Islet2a plays a role in differentiation of another motor neuron subtype-specific characteristic, electrical membrane properties. Our prior work had demonstrated that CaPs have 2.9 and 2.5-fold larger densities of both inward and outward currents, respectively, than do other PMNs [12]. Subsequent work showed that MOs targeting *islet1* had significant effects on the electrophysiological properties of several spinal neuron types [16]. However, in either *islet2a* mutants or morphants, we do not detect any significant differences in CaP inward or outward current densities compared to controls (Figs 2 and 3). Thus, these studies provide no evidence to support a specific role for Islet2a in specifying the >2-fold larger voltage-dependent inward and outward current densities that distinguish CaP from other PMNs.

However, while *islet2a* morphants had motor neuron phenotypes (Figs 4 and 5) similar to those reported previously [15] [17], CaPs and dSMNs in *islet2a* mutants had normal morphologies. It is possible that the *islet2a* T-MO was not sufficiently selective for *islet2a*. However, the primary sequences of the *islet* genes support preferential targeting of *islet2a* over *islet1* or *islet2b* (Table 1; S1 Table). On the other hand, the *islet2a* Ctl MO also had a mild effect on SMN properties (Fig 5).

Discrepant mutant and morphant phenotypes have been reported for an ever-increasing number of genes, raising several caveats about the use of MOs [49] [50] [51] [52] [53] [54]. Explanations to account for MOs not replicating null-mutant phenotypes include off-target/non-specific effects of MOs, unexpected changes in gene expression, and apoptosis via a p53-dependent mechanism [55] [49] [50] [51] [56]. Conversely, gene mutation can lead to compensatory changes in expression [57] [58]. For LIM homeodomain proteins, another consideration is that their stoichiometry in transcriptional complexes influences regulation of downstream genes [59].

Mutants and morphants also differed with respect to gene expression profiles (Fig 6). However, none of the differentially-expressed genes that we identified provided insights into the different motor neuron phenotypes present in mutants compared to morphants. The different developmental stages at which we examined gene expression (48 hpf) and motor neuron morphologies (24 and 72 hpf) may have preempted identification of such genes. It is possible that if we had examined gene expression in 1 dpf embryos and/or just motor neurons, a more informative result would have been obtained.

Our manipulations targeted one member of the Islet gene family, but prior studies suggest that Islet proteins may have redundant functions [15, 47]. We tested whether targeting of *islet2a* either by MOs or gene-editing led to genetic compensation in *islet* gene expression. Consistent with other differentially expressed genes (Fig 6), *islet1* had slightly increased levels in mutants but decreased >2-fold in morphants (Fig 7). The small increase in *islet1* expression detected in 48 hpf mutant transcriptomes might be sufficient to provide genetic compensation, at least at this stage, given that zebrafish Islet1 can substitute for Islet2a and *vice versa* [15].

In contrast, morphants had >2-fold decreased expression levels of *islet1* (Fig 7). The mechanism underlying the changes in *islet* gene expression induced by the T-MO is not obvious. One possibility is that the T-MO may have targeted *islet1* in such a way as to affect expression levels. Arguing against this possibility is the fact that the sequence of the T-MO would be expected to have higher specificity for *islet2a* vs. *islet1* or *islet2b* (Table 1 and S1 Table). However, the Ctl MO also led to a small decrease in *islet1* expression (e.g., Fig 7), suggesting that the T- and Ctl MOs may have had effects not due to targeting of *islet2a*. Regardless of the mechanism(s) underlying the different changes in *islet* gene expression in mutants compared to morphants, the results suggest that targeting *islet2a*, either by MO or gene-editing, leads to changes in expression of *islet1*. Moreover, the effects on *islet1* expression produced by MO compared to gene-editing opposed each other.

An unexpected result was the persistence of reduced, but not abolished, Isl1/2 immunoreactivity in CaPs of 24–28 hpf mutant embryos (Fig 9). Given that we did not detect Isl1/2 expression in the proctodeum (a tissue that expresses *islet2a* but not *islet1*; Fig 1), Islet1 or Islet2b are likely candidates for the Isl1/2 immunoreactive protein detected in CaPs of mutants. In mutants, CaPs may have upregulated either of these genes leading to Isl1/2 immunoreactivity. However, this possibility is not supported by the RNA *in situ* hybridization studies (Fig 8) that did not detect any novel expression of either *islet1* or *islet2b* in CaPs. Segawa et al. [17]also found that overexpression of a dominant-negative Isl2a construct did not lead to ectopic expression of *islet1* in CaPs ((17)-Fig 5B and 5D therein). Another possibility is that there was novel expression of either *islet1* or *islet2b* in CaPs, but at levels lower than can be detected using RNA *in situ* hybridization.

An alternative explanation recalls that all motor neurons normally express Islet1 protein prior to differentiation as different PMN subtypes [8, 10, 60]. It is possible that the turn-over rate of Islet1 protein was sufficiently slow in mutant CaPs to allow persistence of the protein until at least 28 hpf. Further, Hutchinson and Eisen [15] demonstrated that overexpression of *islet1* mRNA rescued spinal neuron phenotypes present in *islet2a* morphants, providing evidence that zebrafish Islet proteins are functionally redundant in the embryonic spinal cord. Similarly, although murine Islet2 mutants lack visceral motor neurons, Thaler et al. [47] proposed that the requirement for Islet2 reflected a need for a specific total concentration of all Islet protein, regardless of identity as Islet1 or Islet2. Given these prior results indicating that Islet1 and Islet2/a can substitute for each other, persistence of an Islet protein in mutant CaPs could have compensated for loss of Islet2a. Distinguishing between these possibilities would need tools not presently available, e.g., antibodies that distinguish between zebrafish Islet1, Islet2a and Islet2b proteins.

In summary, we find no evidence to support a specific, non-redundant role for Islet2a in differentiation of the large inward and outward conductances that distinguish CaP from other PMNs. However, in *islet2a* mutants, CaPs had persistent, albeit reduced, Isl1/2 immunoreactivity. In addition, zebrafish Islet1 and Islet2a proteins can functionally substitute for each other [15]. Thus, we cannot rule out the possibility that function of another (non-Islet2a) Islet protein, during the time when *islet2a* is normally expressed, suffices to specify the large current densities that distinguish CaPs from other PMNs.

## Supporting information

S1 Fig Disruption of Islet2a function using a splice-blocking MO. (A) The *islet2a* splice blocking MO, Sp-MO, targeted the splice junction between intron 1–2 and exon 2. (B) In control (Ctl MO) embryo RNA, RT-PCR amplification of the region spanning exons 1–4 produced a predominant ~800 bp product. RT-PCR using RNA isolated from MO-injected (Sp-MO) embryos yielded two additional bands: *a*, ~1100 bp due to retention of intron 1; *b*, ~600 bp lacking exon 2 (confirmed by DNA sequencing).
(TIF)

S2 Fig RNA *in situ* hybridization reveals *islet2a* expression in a subpopulation of SMNs. (A-D) RNA *in situ* hybridization was performed using transgenic lines that express gfp in either dSMNs (tg(isl1:gfp); A, B) or vSMNs (tg(gata2:gfp); C, D). The red RNA *in situ* hybridization signal for *islet2a* is not detected in gfp^+^ dorsally-projecting SMNs at either 24 (A) or 48 (B) hpf. In contrast, *islet2a* RNA is detected in a subset of ventrally projecting SMNs (C, D). Scale bar in D, for A-D: 25 μm.
(TIF)

S3 Fig Injection of the *islet2a* MO did not alter vSMN or RB electrical membrane properties. (A) Recordings were obtained from vSMN neurons in 48 hpf control (grey trace) vs. morphant (black trace) embryos. No obvious differences were noted in the amplitudes of peak inward or steady-state outward currents between conditions. The voltage protocol used to elicit currents was as described for Fig 3A. (B) The average current densities for net outward and peak inward currents recorded from vSMNs in control (n = 5 cells from 3 embryos) vs. morphant (n = 5 cells from 2 embryos) 24 hpf embryos did not differ. (C) Recordings were obtained from RB neurons in 24 (left) and 48 (right) hpf control (grey traces) and morphant (black traces) *islet2a* embryos. No obvious differences were noted in the amplitudes of peak inward or outward currents between conditions. The voltage protocol used to elicit currents was as described for Fig 3A. (D) The average densities for net outward and peak inward currents recorded from RBs in wildtype vs. morphant embryos did not differ at either 24 (left) or 48 (right) hpf. Sample sizes: 24 hpf– 7 cells from 3 uninjected embryos and 8 cells from 4 T-MO injected embryos; 48 hpf– 11 cells from 3 uninjected embryos and 7 cells from 3 T-MO injected embryos.
(TIF)

S4 Fig Two genes, implicated in regulation of CaP axon morphology, are not differentially expressed between morphant, mutant and controls. qPCR was used to compare expression levels of *nrp1a and plexinA3* between wildtype, mutant, morphant and Ctl MO injected embryos. To facilitate comparisons, expression levels were normalized to that of the control group.
(TIF)

S1 Table MO target and corresponding sequences in *islet1*, *islet2a* and *islet2b*. The sense sequences corresponding to each MO are shown in the top line. For each gene, the intended (*islet2a*) or potential (*islet1*, *islet2b*, *isl1l*) targets corresponding to each MO are shown.
(DOCX)

S1 File RMA file. The excel file presents the RMA normalized data for all 10 samples and all genes.
(XLSX)

S2 File Significant genes for morph vs. mut. The excel file presents the expression levels for 174 genes that were differentially expressed between morph and mut at Q<0.05 and with fold changes that were <-2 or >2.
(XLSX)

S3 File Heat map—Morph vs mut significant genes. The file presents the expression levels for the 174 differentially expressed genes of S2 File and control values as formatted for the heat map of Fig 6B.
(XLSX)

S4 File Significant genes for morph vs. ctl. The excel file presents the expression levels for 201 genes that were differentially expressed between morph and ctl at Q<0.05 and with fold changes that were <-2 or >2.
(XLSX)

S5 File Heat map—Morph vs. ctl significant genes. The file presents the expression levels for the 201 differentially expressed genes of S2 File and control values as formatted for the heat map of Fig 6C.
(XLSX)

S6 File Intersection of morph vs mut and morph vs ctl. The file presents the expression levels for the 64 genes that were differentially expressed between morph and mut as well as between morph and ctl at Q<0.05 and with fold changes that were <-2 or >2.
(XLSX)
