# Supplementary figures and images for: Investigation of Islet2a function in zebrafish embryos: Mutants and morphants differ in morphologic phenotypes and gene expression
Source: PLoS One. 2018 Jun 21;13(6):e0199233. doi: 10.1371/journal.pone.0199233 (PMC6013100; doi:10.1371/journal.pone.0199233)

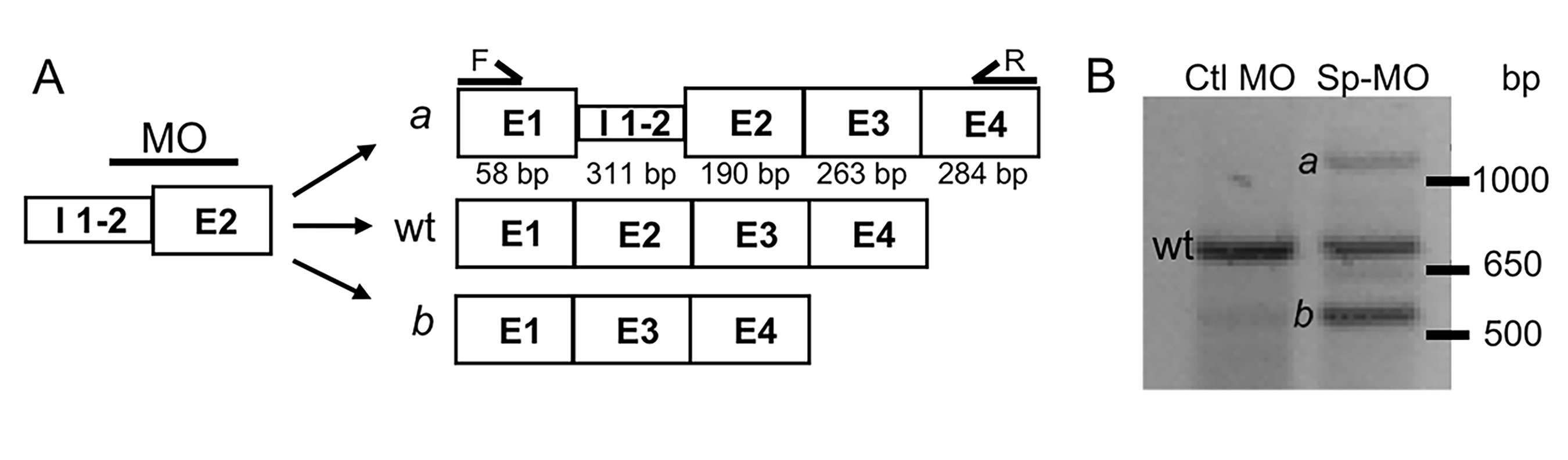

Supplement: S1 Fig — (A) The islet2a splice blocking MO, Sp-MO, targeted the splice junction between intron 1–2 and exon 2. (B) In control (Ctl MO) embryo RNA, RT-PCR amplification of the region spanning exons 1–4 produced a predominant ~800 bp product. RT-PCR using RNA isolated from MO-injected (Sp-MO) embryos yielded two additional bands: a, ~1100 bp due to retention of intron 1; b, ~600 bp lacking exon 2 (confirmed by DNA sequencing). (TIF) [file pone.0199233.s001.tif]

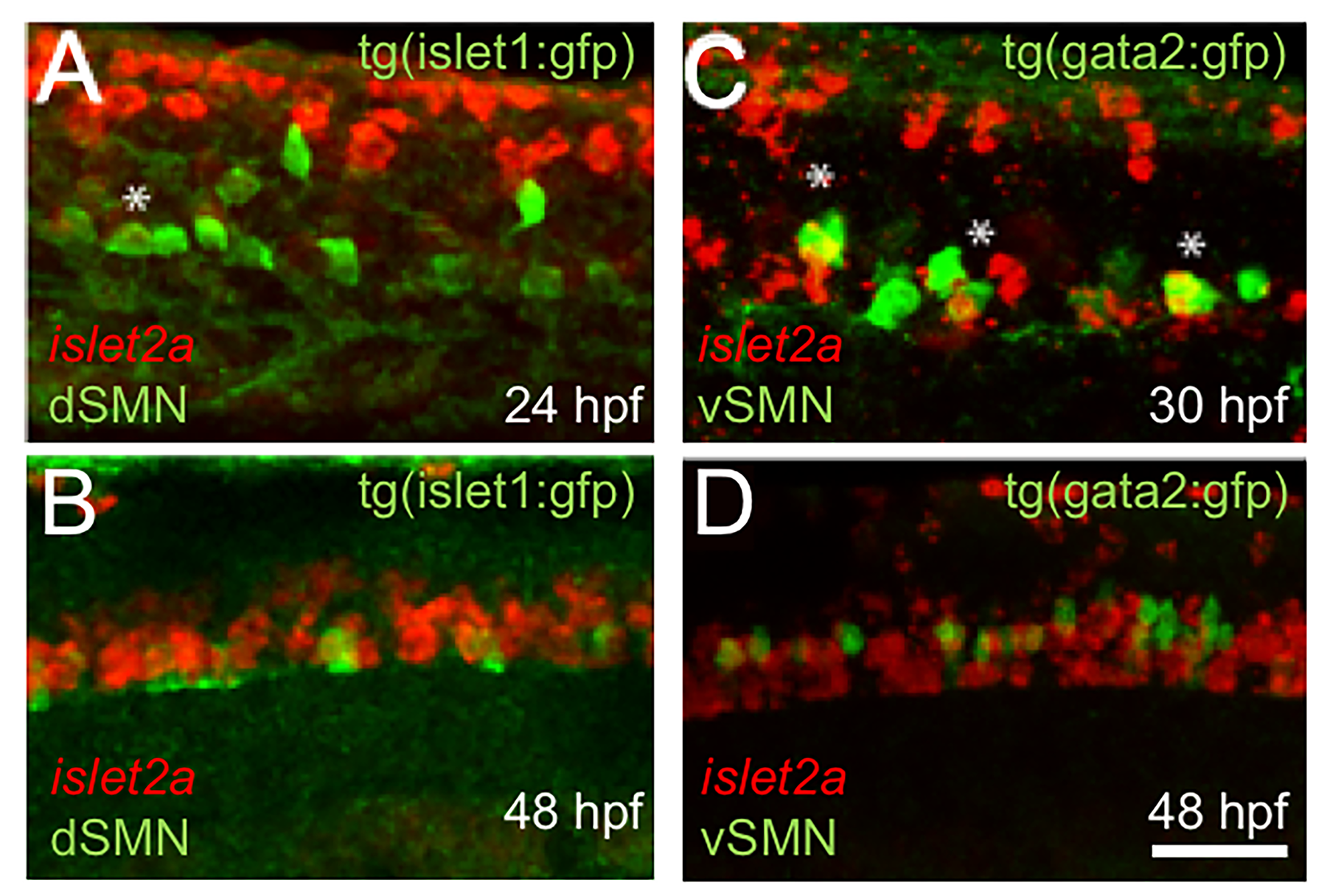

Supplement: S2 Fig — (A-D) RNA in situ hybridization was performed using transgenic lines that express gfp in either dSMNs (tg(isl1:gfp); A, B) or vSMNs (tg(gata2:gfp); C, D). The red RNA in situ hybridization signal for islet2a is not detected in gfp+ dorsally-projecting SMNs at either 24 (A) or 48 (B) hpf. In contrast, islet2a RNA is detected in a subset of ventrally projecting SMNs (C, D). Scale bar in D, for A-D: 25 μm. (TIF) [file pone.0199233.s002.tif]

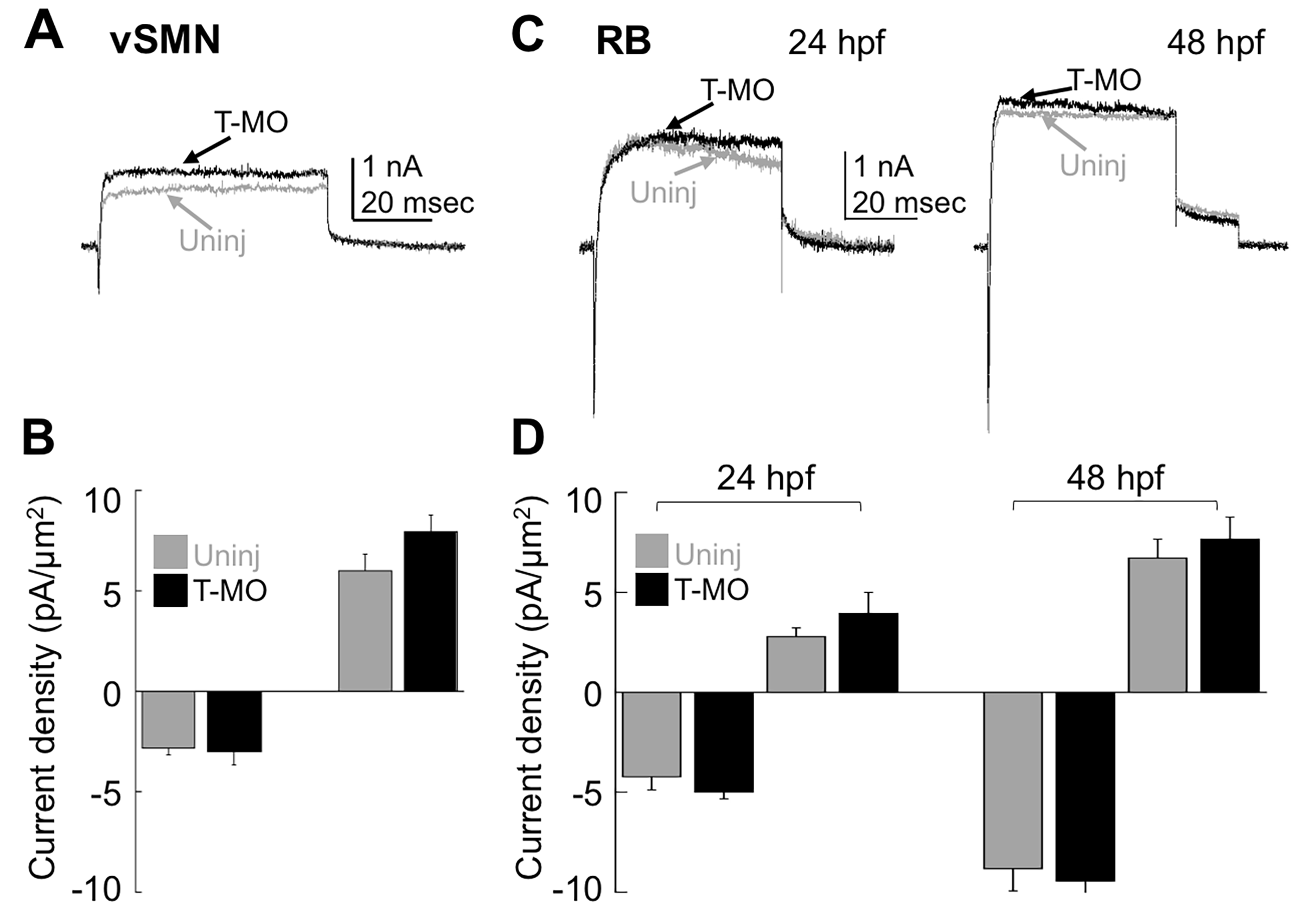

Supplement: S3 Fig — (A) Recordings were obtained from vSMN neurons in 48 hpf control (grey trace) vs. morphant (black trace) embryos. No obvious differences were noted in the amplitudes of peak inward or steady-state outward currents between conditions. The voltage protocol used to elicit currents was as described for Fig 3A. (B) The average current densities for net outward and peak inward currents recorded from vSMNs in control (n = 5 cells from 3 embryos) vs. morphant (n = 5 cells from 2 embryos) 24 hpf embryos did not differ. (C) Recordings were obtained from RB neurons in 24 (left) and 48 (right) hpf control (grey traces) and morphant (black traces) islet2a embryos. No obvious differences were noted in the amplitudes of peak inward or outward currents between conditions. The voltage protocol used to elicit currents was as described for Fig 3A. (D) The average densities for net outward and peak inward currents recorded from RBs in wildtype vs. morphant embryos did not differ at either 24 (left) or 48 (right) hpf. Sample sizes: 24 hpf– 7 cells from 3 uninjected embryos and 8 cells from 4 T-MO injected embryos; 48 hpf– 11 cells from 3 uninjected embryos and 7 cells from 3 T-MO injected embryos. (TIF) [file pone.0199233.s003.tif]

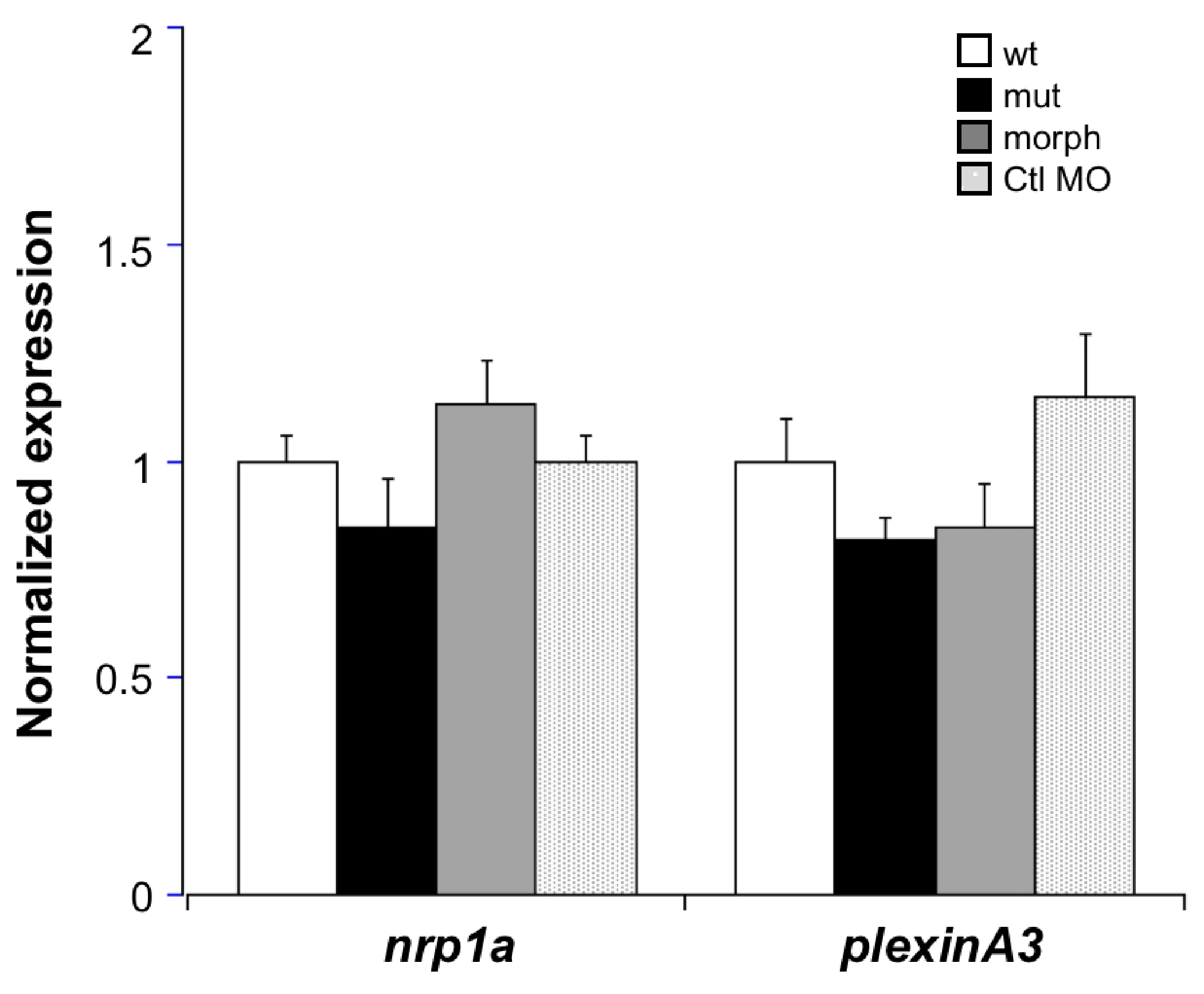

Supplement: S4 Fig — qPCR was used to compare expression levels of nrp1a and plexinA3 between wildtype, mutant, morphant and Ctl MO injected embryos. To facilitate comparisons, expression levels were normalized to that of the control group. (TIF) [file pone.0199233.s004.tif]
