# Supplementary material for: Investigation of Islet2a function in zebrafish embryos: Mutants and morphants differ in morphologic phenotypes and gene expression
Source: PLoS One. 2018 Jun 21;13(6):e0199233. doi: 10.1371/journal.pone.0199233 (PMC6013100; doi:10.1371/journal.pone.0199233)
Supplement: S1 Table — The sense sequences corresponding to each MO are shown in the top line. For each gene, the intended (islet2a) or potential (islet1, islet2b, isl1l) targets corresponding to each MO are shown. (DOCX) [file pone.0199233.s005.docx]

**Table 1: MO specificity for *islet2a***

Number of Mismatches with Target

T-MO Ctl MO Sp-MO

*islet2*  0/25 5/25 0/25

*islet1* 14/25 15/25 12/25

*islet2b* 9/25 10/25 8/25

isl1l 18/25 17/25 19/25
